# Metabolic acidosis: neo-considerations for general surgeons

**DOI:** 10.1308/003588412X13373405387456

**Published:** 2012-05

**Authors:** LCE Martin, U Abah, E Bean, S Gupta

**Affiliations:** East and North Hertfordshire NHS Trust,UK

**Keywords:** Metabolic Acidosis, Urinary Diversion, Associated Conditions

## Abstract

Hyperchloraemic metabolic acidosis is a documented complication of neobladder formation. However, it usually improves with time and is mild. Severe and persistent metabolic acidosis may manifest when patients undergo further surgery for other reasons. Neobladder formation following radical cystectomy or cystoprostatectomy is becoming increasingly more common, and surgeons treating patients with neobladders should recognise and treat metabolic acidosis with intravenous fluids and bicarbonate.

## Case history

An 80-year-old man was admitted from clinic 6 weeks following a right hemicolectomy for a Dukes’ B (pT3N0MX) caecal adenocarcinoma of the caecum, with a simultaneous cholecystectomy and incisional hernia repair. He reported a significant deterioration since discharge with multiple daily loose motions, lethargy and 19kg weight loss. His medical history included asbestosis, interstitial lung fibrosis, cystoprostatectomy, incisional hernia and gallstones. Pre-operatively, he lived independently and had good exercise tolerance.

Examination revealed gross dehydration, weakness and hypotension (50mmHg/30mmHg), which improved with fluid resuscitation. The patient’s abdomen was soft and non-tender with an intact midline laparotomy repair. Blood analysis revealed: haemoglobin 10.5g/dl, white cell count 8 × 10^9^/l, C-reactive protein <5mg/l, creatinine 183umol/l, urea 18.5mmol/l and normal liver function tests. Arterial blood gas revealed: pH 7.17, lactate 1.43mmol/l, sodium 125mmol/l, potassium 3.06mmol/l, calcium 1.30mmol/l, base excess -13.5mEq/l, chloride 114mmol/l and bicarbonate 14.3mmmol/l. Hyperchloraemic, hypokalaemic metabolic acidosis was presumed. Computed tomography of the abdomen was unremarkable and stool culture was negative.

A diagnosis of malnutrition, dehydration and acute renal failure was made. Treatment entailed intravenous fluids, cholestyramine, loperamide and a high calorie oral intake. There was a fast clinical recovery and renal function returned to normal within 48 hours although a hyperchloraemic metabolic acidosis persisted for two weeks. This appeared unexplained until the patient’s past medical history was considered in detail, which included a radical cystoprostatectomy and Studer ileal neobladder formation 11 years previously for G3pT2 transition cell carcinoma of the bladder.

## Discussion

Urinary diversion via different types of bowel reservoir is becoming increasingly more common in patients who have undergone a radical cystectomy or cystoprostatectomy. Currently, around 10% patients in the UK undergo orthotropic bladder reconstruction after cystectomy but this figure is rising rapidly. Orthotopic bladder reservoir should be considered the gold standard with which other forms of urinary diversion are compared.^1^ Neobladder using an ileal segment is the most common. However, colonic segments are also used. As the number of patients with neobladders continues to rise, general surgeons and physicians need to be more aware of the complications that may present to them. Without a conduit or obvious stoma, a neobladder can be forgotten until complications arise. General surgeons need to be aware of the implications a neobladder may have on their planned or emergency surgery, especially the metabolic implications. A urology consultation is often useful prior to embarking on major surgery.

Metabolic acidosis following ileal neobladder is a recognised phenomenon but suitably rare that many surgeons have not encountered it. Metabolic acidosis has been reported as having an incidence of between 4% and 65% in reconstructed bladders, and this varies between the segments of bowel and techniques used.^2^ It is usually a hyperchloraemic metabolic acidosis. It is frequently mild in its manifestations although it may become symptomatic and severe when compounded by renal insufficiency owing to dehydration, and, in this case, by diarrhoea after a right hemicolectomy. Renal function has been highlighted as a significant influential factor in metabolic disturbance in the presence of orthotopic bladders.^2^ Surgeons need to be aware of the impact that renal function and concomitant illness may have on the orthotopic bladder.

Typical symptoms of significant metabolic acidosis include lethargy, fatigue, dehydration and weight loss. Acidosis and hyperchloraemia result from excessive chloride absorption from urine pooled in the intestinal segment.^3^ The absorption of urea seems to have little clinical significance. This process increases with prolonged exposure of the concentrated urine to the intestinal mucosa. This is often the case in dehydration where urine is more concentrated and may remain in the neobladder segment for longer. Hydration is therefore paramount in patients with neobladders. Diluted urine will ensure a low concentration of electrolytes in the urine and reduce the length of time the urine remains in contact with the absorbing surface. These factors were highlighted by Eiseman and Bricker in 1952.^3^

Metabolic acidosis is more common in the early post-operative period. However, it may persist. Martínez-Cornelio *et al* reported metabolic acidosis as the most frequent early complication of neobladder formation in their centre, demonstrated in 66% of patients.^4^ In another case series, Neuzillet *et al* reported a 15% complication rate of metabolic acidosis and all of these cases responded well to oral medication.^5^ In a large study involving 1,000 neobladders, Hautmann *et al* found that 70% of patients had metabolic acidosis in the early post-operative period, which was easily treated with sodium bicarbonate.^6^ As time progressed, the requirement for bicarbonate reduced and only 33% of patients required bicarbonate one year post-operatively.^6^

Hautmann *et al* also commented on severe cases of metabolic acidosis requiring hospital admission owing to hypovolaemia and salt losing acidotic state (11 of 923 patients).^6^ These patients presented in a similar way to the patient in our report. They were treated with oral sodium bicarbonate and lactated Ringer’s solution together with catheterisation in order to decrease urine contact time with the bowel mucosa.

Kristjánsson *et al* investigated the influence of the glomerular filtration rate (GFR) on electrolyte and acid–base homeostasis in patients with neobladders (colonic segments). Under normal circumstances the effects of increased proton absorption can be compensated for with a GFR of >55ml/min/1.37m^2^.^7^ However, in our case, owing to the impact of major abdominal surgery and diarrhoea, the GFR dropped to 31ml/min/1.37m^2^.

As well as metabolic acidosis, the other documented complications of neobladder include hydronephrosis, incisional hernia, ileus or small bowel obstruction, neobladder rupture and peritonism, local recurrence and chronic diarrhoea.^6^

## Conclusions

Metabolic acidosis can be a serious early or late complication of neobladder formation and may complicate other surgery. In patients with neobladders undergoing major surgery, extra care should be taken to ensure that metabolic acidosis does not compromise outcomes. Any surgeon treating a patient with a neobladder should be aware of the associated complications and should monitor acid–base balance with regular blood tests, treating with bicarbonate if necessary. Urologists should be involved in the management and surgeons should consider urological input during surgery to ensure no injury occurs to the vascular pedicle of the reconstructed urinary tract.
